# Editorial: Breaking the cycle: attacking the malaria parasite in the liver

**DOI:** 10.3389/fmicb.2015.00810

**Published:** 2015-08-07

**Authors:** Ute Frevert, Urszula Krzych, Thomas L. Richie

**Affiliations:** ^1^Division of Medical Parasitology, Department of Microbiology, New York University School of MedicineNew York, NY, USA; ^2^Cellular Immunology, Walter Reed Army Institute of ResearchSilver Spring, MD, USA; ^3^Sanaria Inc.Rockville, MD, USA

**Keywords:** *Plasmodium*, immunity, antigen-presenting cell, B cell, CD8 T cell, CD4 T cell, vaccine, adjuvant

*Plasmodium falciparum* malaria remains one of the most serious health problems globally. Immunization with attenuated parasites elicits multiple cellular effector mechanisms capable of eliminating *Plasmodium* from the liver. However, malaria liver stage immunity is complex. The anatomic site of priming of naive *Plasmodium*-specific CD8 T cells, be it in the lymph nodes draining the site of *Plasmodium* antigen deposition by the mosquito or in the liver, may in fact determine the specificity of the effector CD8 T cells. The participation of particular antigen-presenting cells (Corradin and Levitskaya, 2014) and tissue signatures that influence the activation of intrahepatic CD8 T cells against malaria sporozoites (Morrot and Rodrigues, 2014) are still incompletely understood. Similarly, how effector CD8 T cells detect the few infected hepatocytes in the large liver and the mechanisms they use to kill the intracellular parasites are unknown. The unique immunological properties of the liver could explain why effector CD8 T cells are so inefficient in finding and eliminating the hepatic stages of *Plasmodium* (Bertolino and Bowen, 2015). Effector CD8 T cells require help from CD4 T cells, and antigen-presenting cells are thought to stimulate CD4 T cell licensing and enhance their capacity to optimally activate CD8 T cells (Crispe, 2014). Helper CD4 T cells also aid in the development of B cell-mediated immunity (Dups et al., 2014). High levels of interstitial antibodies can immobilize sporozoites in the skin and circulating antibodies can prevent the parasites from infecting the liver (Vanderberg, 2015). A better understanding of fine specificity and quantities of antibodies required for protection and the antigens recognized by neutralizing antibodies will facilitate the design of refined malaria vaccines that induce robust, long-lived, protective B cell responses (Dups et al., 2014).

Animal models have traditionally played an important role in the discovery of the basic parameters of CD8 T cell-mediated immunity, as ethical and practical limitations preclude study of the cellular and molecular mechanisms by which malaria vaccines induce protection in humans. Highlighting the complexity of *Plasmodium* liver stage immunity, comparison of murine malaria models led to the identification of protective memory CD8 T cell responses that differed quantitatively and qualitatively, depending on the *Plasmodium* species (Van Braeckel-Budimir and Harty, 2014).

The mechanisms effector CD8 T cells use to recognize and eliminate *Plasmodium* from the liver are also unknown. Adoptive transfer of circumsporozoite protein-specific CD8 T cells into transgenic mice that express matching MHC class I molecules either exclusively on hepatocytes or on dendritic cells suggests that recognition of hepatocytes is sufficient to confer protection (Huang et al., 2015). However, the formation of immunological synapses between T cells and hepatocytes has not been observed in the intact liver suggesting that classical granule-mediated cytotoxicity is dispensable for parasite killing. In fact, mounting evidence suggests that effector CD8 T cells elicited by attenuated sporozoite vaccines recognize a subpopulation of hepatic dendritic cells and use cytokines to eliminate the parasites at a distance, without direct contact with infected hepatocytes (Frevert and Krzych, 2015). This model is supported by the lymphogenic features of the liver (Frevert and Krzych, 2015). The contribution of Kupffer cells and other liver-resident and recruited antigen-presenting cells to the effector phase against *Plasmodium* likely varies with the immune status of the host (Bertolino and Bowen, 2015; Frevert and Krzych, 2015).

The observation that both antigen-specific and antigen-unrelated CD8 T cells cluster around infected hepatocytes led to the proposal that antigen-specific effector CD8 T cells recruit other T cells to the site of infection and that the resulting inflammatory microenvironment augments parasite killing by antigen-specific and antigen-unrelated bystander cells (Bayarsaikhan et al., 2015). The significance of antigen-dependent focal inflammation and its consequences for the elimination of the intracellular parasites is discussed (Fernandes et al., 2014).

Unlike *P. falciparum* and rodent species, *P. vivax* forms dormant liver stages that may relapse weeks, months, or years after the primary infection, leading to new bouts of illness. Non-human primate model systems have been developed to study the immunobiology of the relapse phenomenon and to screen for biomarkers for *P. vivax* and a related simian parasite (Joyner et al., 2015).

The liver is also a central player in the defense against *Plasmodium* blood stages. Activation of Toll-like receptors (TLRs), acute phase proteins, phagocytic activity, and cytokine-mediated pro- and anti-inflammatory responses are all part of the liver-inherent immune system (Wunderlich et al., 2014).

Efforts to develop a successful malaria vaccine have been the focus of substantial research activities for decades. Immunization with live-attenuated sporozoites can elicit sterile immunity. Whether attenuation is achieved by irradiation or genetic modification, CD8 T cells play an essential role in the resulting sterilizing protection in several experimental models. Inoculation of infectious sporozoites under chemoprophylaxis also confers long-lasting sterile protection against homologous parasite strains in humans, although the exact mechanism is unclear. Chloroquine, the first drug used for chemoprophylaxis, neither eliminates *Plasmodium* liver stages nor delays parasite development (Sahu et al., 2015). Anti-malarial compounds such as quinine, quinones, and resveratrol, which may be present in the diet of individuals living in some endemic areas, are thought to contribute to protection against blood stage infection (Dalai et al., 2015).

Unlike whole organism vaccines, most subunit vaccine candidates fail to induce substantial and lasting protection. Novel approaches are therefore under investigation to identify antigens responsible for protection against the different parasite stages (Chia et al., 2014). These include the mining of genomic, proteomic and transcriptomic datasets to rationally identify immunological signatures associated with more potent immunity than occurs after natural exposure (Proietti and Doolan, 2014). Another focus is on novel non-inflammatory nanoparticle-based adjuvants, which induce high CD8 T cell responses without expanding myeloid-derived suppressor cells or inflammation-reactive Tregs at the site of priming (Wilson et al., 2015).

We hope that this Frontiers eBook offers insight into the many efforts aiming at breaking the life cycle of *Plasmodium* in the liver. A more thorough understanding of the mechanisms leading to sterile protection is a prerequisite for developing a malaria vaccine that protects the 40% of the world's population at risk of infection. We thank all contributing authors for bringing a broad range of expertise to this Frontiers Topic.

## Conflict of interest statement

The authors declare that the research was conducted in the absence of any commercial or financial relationships that could be construed as a potential conflict of interest.
